# Molecular Dynamics Simulations Suggest that Electrostatic Funnel Directs Binding of Tamiflu to Influenza N1 Neuraminidases

**DOI:** 10.1371/journal.pcbi.1000939

**Published:** 2010-09-23

**Authors:** Ly Le, Eric H. Lee, David J. Hardy, Thanh N. Truong, Klaus Schulten

**Affiliations:** 1 Beckman Institute, University of Illinois at Urbana-Champaign, Urbana, Illinois, United States of America; 2 Department of Chemistry, University of Utah, Salt Lake City, Utah, United States of America; 3 School of Biotechnology, Ho Chi Minh International University and Saigon Institute for Computational Science and Technology, Ho Chi Minh City, Vietnam; 4 Center for Biophysics and Computational Biology, University of Illinois at Urbana-Champaign, Urbana, Illinois, United States of America; 5 College of Medicine, University of Illinois at Urbana-Champaign, Urbana, Illinois, United States of America; 6 Department of Physics, University of Illinois at Urbana-Champaign, Urbana, Illinois, United States of America; University of California, Irvine, United States of America

## Abstract

Oseltamivir (Tamiflu) is currently the frontline antiviral drug employed to fight the flu virus in infected individuals by inhibiting neuraminidase, a flu protein responsible for the release of newly synthesized virions. However, oseltamivir resistance has become a critical problem due to rapid mutation of the flu virus. Unfortunately, how mutations actually confer drug resistance is not well understood. In this study, we employ molecular dynamics (MD) and steered molecular dynamics (SMD) simulations, as well as graphics processing unit (GPU)-accelerated electrostatic mapping, to uncover the mechanism behind point mutation induced oseltamivir-resistance in both H5N1 “avian” and H1N1pdm “swine” flu N1-subtype neuraminidases. The simulations reveal an electrostatic binding funnel that plays a key role in directing oseltamivir into and out of its binding site on N1 neuraminidase. The binding pathway for oseltamivir suggests how mutations disrupt drug binding and how new drugs may circumvent the resistance mechanisms.

## Author Summary

Oseltamivir (Tamiflu) is the main antiviral drug used to fight viral influenza outbreaks such as the recent swine flu (H1N1pdm) global pandemic and avian (H5N1) outbreak in Asia. Oseltamivir inhibits a protein on the surface of flu viruses called neuraminidase, which is responsible for releasing newly formed viruses. The rapid emergence of drug resistance in H5N1 avian flu (and recently the H1N1pdm strain) has already motivated numerous studies to understand how mutations render oseltamivir ineffective, but no focused investigation has yet elucidated the specific mechanism behind mutation-induced drug resistance. Here, large scale computer simulations are employed to study both H5N1 and H1N1pdm neuraminidase to answer the questions: how does N1-subtype neuraminidase bind oseltamivir, and how would mutations alter this process? The key finding revealed in our simulations is the discovery of oseltamivir binding to neuraminidase by a charged pathway on the protein surface. We suggest that point mutations may disrupt drug binding by interfering with this pathway. Our results explain the fundamental mechanism behind oseltamivir resistance and pave the way for the design of drugs that circumvent viral drug resistance.

## Introduction

Oseltamivir, better known by its commercial name Tamiflu, is currently the most important antiviral drug employed to combat the flu virus [1]. Oseltamivir functions by competitively binding, against a natural substrate on cells called sialic acid (SA), to a flu protein called neuraminidase N1 subtype, which is responsible for mediating the release of newly synthesized virion particles from an infected cell [2]. Of grave concern however, is the emergence of oseltamivir-resistant strains of N1-subtype influenza (including H5N1 [3], seasonal H1N1 [4], and H1N1pdm [5]–[7]). Understanding the mechanism behind mutation-induced drug resistance in neuraminidase N1 subtype is critical for the development of effective therapies.

The rapid emergence of oseltamivir resistance in H5N1 avian flu has motivated already numerous studies, both experimental and theoretical, to uncover how point mutations to neuraminidase alter drug binding [8]–[12]. The recent elucidation of crystal structures for both wildtype and mutant H5N1 neuraminidases have opened up a door for an investigation of drug resistance mechanisms and structure-based drug design at the atomic level [12], [13]. From these structures it has been suggested that oseltamivir resistance due to point mutations arise from a destabilization of the hydrophobic packing that binds oseltamivir tightly within the neuraminidase active site [12]. Crystal structures represent a time frozen snapshot into a possible conformation of drug-protein interaction. Drug binding, however, is a dynamic process and computational studies used the crystal structures as starting points to shed light on exactly how protein flexibility and point mutations influence drug-protein endpoint interactions [8]–[11]. Despite these initial inroads of studies based on molecular dynamics (MD) simulations, the current understanding of the mechanism behind drug resistance remains incomplete and some conclusions are conflicting. For example, in one study [10] it was reported that the H274Y mutation disrupts E276-R224 salt bridges that accommodate the hydrophobic pentyl group of oseltamivir, while in another study [8] the same salt bridges were observed to be stable. Up to this point, all proposed mechanisms for oseltamivir resistance have focused mainly on effects of mutations on the SA binding site and equilibrium drug binding affinities.

Since neither H274Y nor N294S are active site mutations [14], studies which only focus on end-point interactions between drug and protein are unable to elucidate if these mutations impact the actual drug binding process, i.e., affect binding kinetics. In this study, we not only employ molecular dynamics (MD) on oseltamivir-bound forms of wildtype and known drug resistant mutants (H274Y, N294S) of avian H5N1 and swine H1N1pdm neuraminidases, but also steered MD (SMD) simulations on avian H5N1 to investigate oseltamivir binding/unbinding pathways. The electrostatic potentials calculated reveal a distinct negatively charged column of residues, bridging the SA binding site and the edge of the binding cavity mouth, that apparently functions as a drug binding/unbinding funnel. Oseltamivir is observed to diffuse, in our simulations, into and out of the neuraminidase active site via this funnel. During drug passage, our simulations did not reveal any specific interactions beyond an obvious electrostatic attraction between drug and protein, suggesting a binding pathway governed by an electrostatic funnel. We also suggest a role that the drug resistant mutations H274Y and N294S play in disrupting this funnel and in altering the binding process between oseltamivir and N1-subtype neuraminidase. Given that the drug binding rates have been found to be significantly diminished for H5N1 mutants [12], it is possible that the stated mutations (H274Y, N294S) induce drug resistance by disrupting the drug binding kinetics as well as by active site endpoint interactions affecting binding affinity. We suggest that our observations regarding the oseltamivir binding behavior of H5N1 should apply for H1N1pdm (associated with the recent pandemic), since the two proteins have very high sequence identity (91.47%) and share a conserved drug binding site.

## Results

The following results are based on simulations, summarized in Table 1 (see Methods section), of drug bound N1-subtype neuraminidases, including avian H5N1 and H1N1pdm, both as wildtype and as two oseltamivir-resistant mutant systems. The individual simulations will be referred to by the designations listed in the “Name” column of Table 1. We first describe interactions that stabilize oseltamivir in six neuraminidase systems observed from equilibrium (EQ) simulations (*simEQ1–6*). Then we present our observations of electrostatics calculated from these simulations. Next, we discuss characteristics of drug binding and unbinding seen in both steered molecular dynamics (SMD) (*simSMD1*) and subsequent relaxation simulations (*simFEQ1–10*). Finally we relate the results of our simulations to oseltamivir resistance.

**Table 1:** Summary of simulations.

Name	Structure	Atoms	Water	Type	Ensemble	Time (ns)
simEQ1	H5N1+oseltamivir	34860	9670	EQ	NpT	40
simEQ2	H1N1pdm+oseltamivir	34707	9604	EQ	NpT	40
simEQ3	H274Y-H5N1+oseltamivir	34880	9670	EQ	NpT	40
simEQ4	H274Y-H1N1pdm+oseltamivir	34729	9604	EQ	NpT	40
simEQ5	H294S-H5N1+oseltamivir	34873	9670	EQ	NpT	40
simEQ6	N294S-H1N1pdm+oseltamivir	34727	9604	EQ	NpT	40
simSMD1	H5N1+oseltamivir	34860	9670	SCV	NV	15
simSMD2	H5N1+oseltamivir	34860	9670	SCV	NV	10
simSMD3	H5N1+oseltamivir	34860	9670	SCV	NV	5
simFEQ1	H5N1+oseltamivir	34860	9670	EQ	NpT	15
simFEQ2	H5N1+oseltamivir	34860	9670	EQ	NpT	10
simFEQ3	H5N1+oseltamivir	34860	9670	EQ	NpT	15
simFEQ4	H5N1+oseltamivir	34860	9670	EQ	NpT	50
simFEQ5	H5N1+oseltamivir	34860	9670	EQ	NpT	100
simFEQ6	H5N1+oseltamivir	34860	9670	EQ	NpT	10
simFEQ7	H5N1+oseltamivir	34860	9670	EQ	NpT	15
simFEQ8	H5N1+oseltamivir	34860	9670	EQ	NpT	50
simFEQ9	H5N1+oseltamivir	34860	9670	EQ	NpT	50
simFEQ10	H5N1+oseltamivir	34860	9670	EQ	NpT	50

The “Structure” column lists the type of neuraminidase, associated mutations, and drug modeled in each system. The “Atoms” and “Water” columns show total number of atoms, and number of water molecules, respectively. The “Ensemble” column lists the variables held constant during simulations; N, p, T, and V correspond to number of atoms, pressure, temperature, and volume, respectively. Under “Type”, EQ denotes equilibration, and SCV denotes constant velocity SMD simulation with a velocity of 0.05, 0.10, and 0.25 Å/ps, for *simSMD1*, *simSMD2*, *and simSMD3*, respectively. For *simEQ1* to *simEQ6*, the simulation times involve a 20 ns setup/equilibration run followed by a 20 ns production run. *SimSMD1* is a steered MD simulation with the starting structure from equilibrated *simEQ1* (“locked” drug position). In *simFEQ1* to *simFEQ10*, the starting structure is a bound, rotated position of oseltamivir from *simSMD1* after 7.5ns of simulation, when several of the drug's stabilizing hydrogen bonds to the protein are ruptured.

### Oseltamivir binds to a conserved hydrogen bond network

Equilibrium simulations of six oseltamivir-neuraminidase complexes were carried out, including avian H5N1 and swine flu H1N1pdm proteins, for one wildtype (WT) neuraminidase (*simEQ1–2*) and two mutants, namely, H274Y (*simEQ3–4*) and N294S (*simEQ5–6*). The root mean square deviations (RMSD) of the proteins shown in Figure S1 demonstrate the stability of the simulated models and the RMSDs of oseltamivir in Figure S2 and Figure S3 show that the drug, over 20 ns, binds stably to the SA binding pockets for both WT and mutants (figures and text labeled “S” are in Supplementary Materials).

*SimEQ1–6* reveal that hydrogen bonds form the bulk of the interactions which stabilize oseltamivir in the SA binding pocket (see Text S1 for a discussion of protein stability and Text S2 for detailed discussion of hydrogen bond networks). In both wildtype and mutant simulations, hydrogen bonds are well conserved between oseltamivir and binding site residues E119, D151, R292, and R371. Specifically, R292 and R371 are observed to hydrogen bond with oseltamivir's carboxylate moiety, and E119 and D151 with oseltamivir's amino group. The H274Y mutation, however, is seen to disrupt the hydrogen bonding of oseltamivir's acetyl group with R152, a stabilizing interaction in the wildtype and N294S systems. The carboxylate group of oseltamivir exhibits a weak hydrogen bond with Y347 of the avian H5N1 neuraminidases (Figure S4), which corresponds to the N347 mutation in the H1N1pdm proteins (Figure S5). Histograms listing the frequency of hydrogen bonds between oseltamivir and neuraminidases are shown in Figure S4A and Figure S5A, with schematic views of the specific residues involved in the drug-protein hydrogen bond pairings provided in Figure S4B, Figure S4C, and Figure S4D for *simEQ1*, *simEQ3*, and *simEQ5*, respectively, and in Figure S5B, Figure S5C, and Figure S5D for *simEQ2*, *simEQ4*, and *simEQ6*, respectively. In the case of the H274Y mutant, we observed a loss of the hydrogen bond between oseltamivir and R152, as well as a decrease in favorable hydrophobic packing between oseltamivir's pentyl group and neuraminidase's hydrophobic subsite (I222-R224-A246-E276). Figure S6, illustrating an increase in solvent accessible surface area (SASA) for the H274Y systems, is discussed in Text S3, along with further description of oseltamivir's hydrogen bonding network observed in our simulations as compared to previous studies [8], [12]. While the H274Y mutant clearly showed decreased binding stability for oseltamivir's pentyl group within the SA binding pocket, disruption to endpoint interactions alone within simulation timescales may not cover all possible effects of the two non-active site mutations H274Y and N294S, on oseltamivir binding. Therefore we turn our attention to other physical characteristics which may also govern drug binding kinetics.

### The electrostatic potential of neuraminidases suggests a binding pathway for oseltamivir

The electrostatic potential of neuraminidases serves as an important driving force both to direct the diffusion of ligands into the SA active site [15], [16] and to stabilize the end point interactions between ligand and the proteins [17], [18]. For example, computational studies investigating the quantitive free binding energy associated with neuraminidase inhibitor binding reveal that the local electrostatic potential of the drug binding pocket significantly impacts the final binding pose and stability of the drug [18]. In regard to neuraminidase, a previous study [16] using Brownian dynamics simulations suggested that electrostatic steering guides SA and neuraminidase inhibitors to enter the primary SA active site of N1 neuraminidase via a secondary SA binding site proximal to loop-430 [15]. A second, well known example in which a cationic substrate is drawn through a short channel to enter a narrow active site due to electrostatic steering is seen in the case of acetylcholinesterase [19], [20]. Even though a possible role of the electrostatic potential for drug binding in neuraminidases had been discussed before, the extensive electrostatic calculations required for fully characterizing the role of electrostatic steering had not yet been carried out.

**Figure 1:**
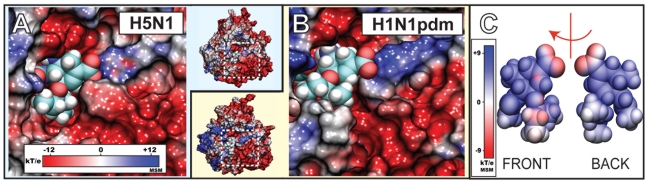
Electrostatic surface potential of the sialic acid (SA) binding pocket of H1N1pdm and oseltamivir. Shown in A) and B) are closeup views of the SA binding pocket with drug bound H1N1pdm and avian H5N1 neuraminidase, respectively. The region of the binding pocket, where the drug binds, exhibits a negative potential (colored red), whereas the opening of the pocket is surrounded by a highly positive potential ring (colored blue). C) illustrates the electrostatic potential around oseltamivir. Shown are the “front” side facing the annulus of the binding pocket and the “back” side facing the interior of the binding pocket. Simulations *simEQ1* through *simEQ6* revealed the presence of a negatively charged funnel at the mouth of the binding pocket (colored in red) which may play a role in oseltamivir binding and unbinding. The electrostatic surface potentials were generated using the multilevel summation method (MSM) with color scale bars shown in insets.

In order to address the shortcoming, the electrostatic surface potentials of the equilibrated systems were calculated and averaged across every trajectory frame in *simEQ1–6* employing GPU-accelerated multilevel summation [21] (see Methods). The resulting electrostatic maps are shown in Figure 1A for H5N1 and in Figure 1B for H1N1pdm. The maps reveal that the binding pocket possesses a negative potential (colored red), and that it is surrounded by a positive potential ring (colored blue). The electrostatic surface potentials of the neuraminidases simulated show, in particular, a column of negatively charged residues that form a pathway, 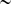
10Å in length, between the primary SA binding site and the mouth of the binding cavity. Since oseltamivir itself has a positive electrostatic surface potential, as illustrated in Figure 1C, the question arises whether the negatively charged surface column in N1 neuraminidases plays a role in the binding kinetics of oseltamivir. To answer this question, and following insight from earlier studies of binding processes [22]–[25], we employed simulations (described in Methods) to pull oseltamivir out of the SA binding site and probed unbinding in order to reveal actually the properties of the binding pathway.

### Studying drug unbinding through SMD simulations to learn about drug binding

To simulate binding of oseltamivir would be the most natural approach to identify the drug binding pathway. Unfortunately, the needed computations are impossible since the duration of binding is too long. Unbinding enforced through external forces in so-called steered MD (SMD) simulations requires much less time such that the calculations are feasible. Fortunately, unbinding simulations can reveal features characteristic for the reverse process of binding, as demonstrated many times before, e.g., in [22]–[25]. We note, however, that while SMD simulations may reveal potential entry and exit pathways for drug binding, they do not provide information regarding the thermodynamic feasibility of binding (which correlates to drug inhibition power) except when used with sampling methodologies [25]–[27] not applied in the present case.

**Figure 2:**
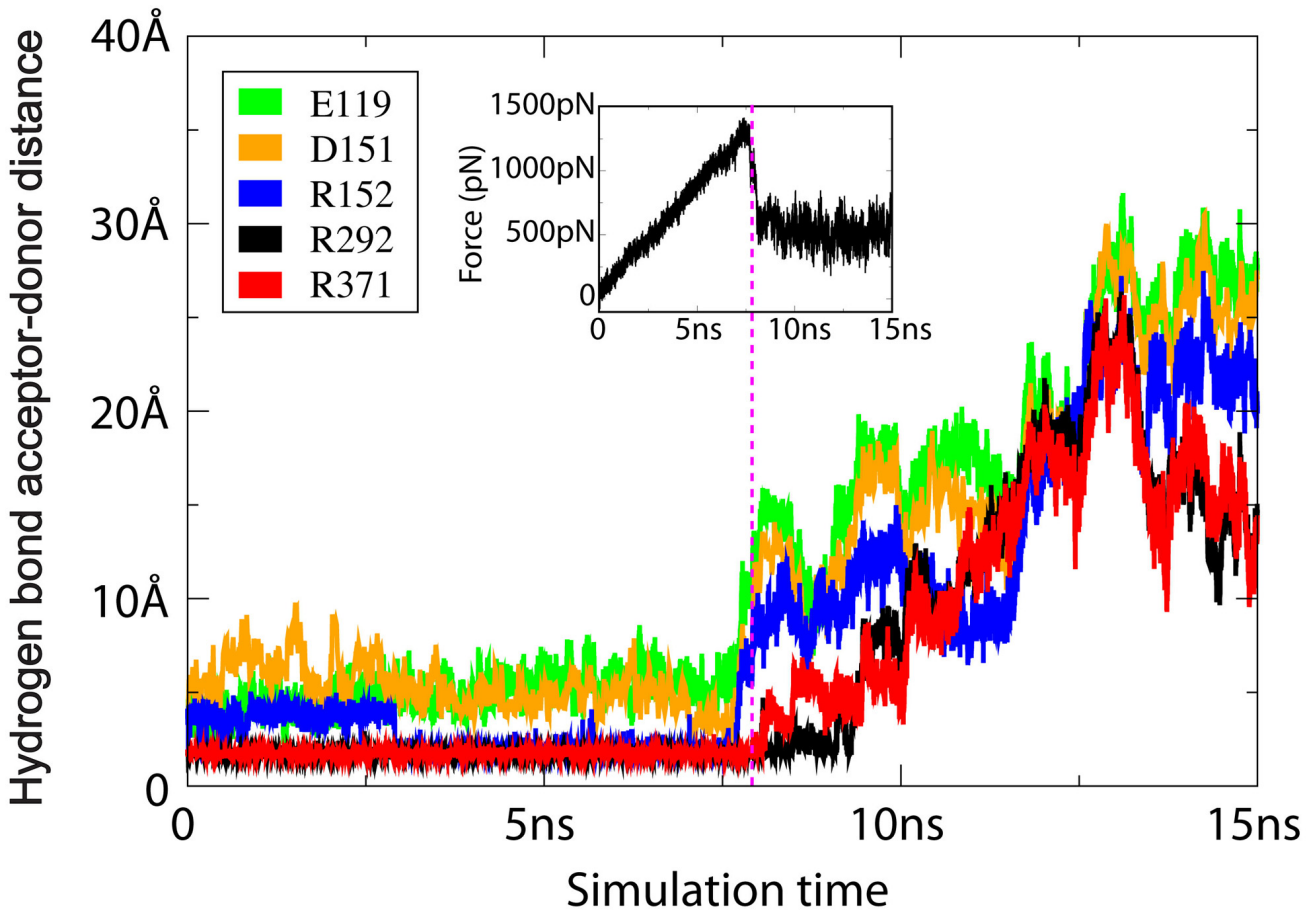
Distances between hydrogen bond acceptor-donor pairs between oseltamivir and sialic acid (SA) binding pocket amino acids vs. simulation time in *simSMD1*. Most hydrogen bonds are quickly broken by the pulling force except for those with R292 and R371, which fully rupture only after 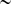
8 ns, corresponding to the peak of the curve of the applied force vs. simulation time (shown in inset).

In *simSMD1*, a pulling force was applied to rupture all stabilizing hydrogen bonds between H5N1 and oseltamivir, and draw the drug away from the SA binding site. The results of *simSMD1* show that the response of oseltamivir to the pulling force evolves in three distinct stages: 1) from 0 to 8 ns, a buildup of force during which hydrogen bonds between oseltamivir with E119, D151 and R152 are ruptured; 2) at 8 ns when the remaining stable hydrogen bonds with R292 and R371 rupture; 3) after 8 ns when the drug is pulled out of the binding pocket. Figure 2 shows the force dependent rupture of hydrogen bonds in *simSMD1* presenting both hydrogen bond length and (in the inset) the force vs. time curve. We carried out simulations at different pulling velocities (*simSMD2–3*) that all exhibited similar unbinding behavior.

**Figure 3:**
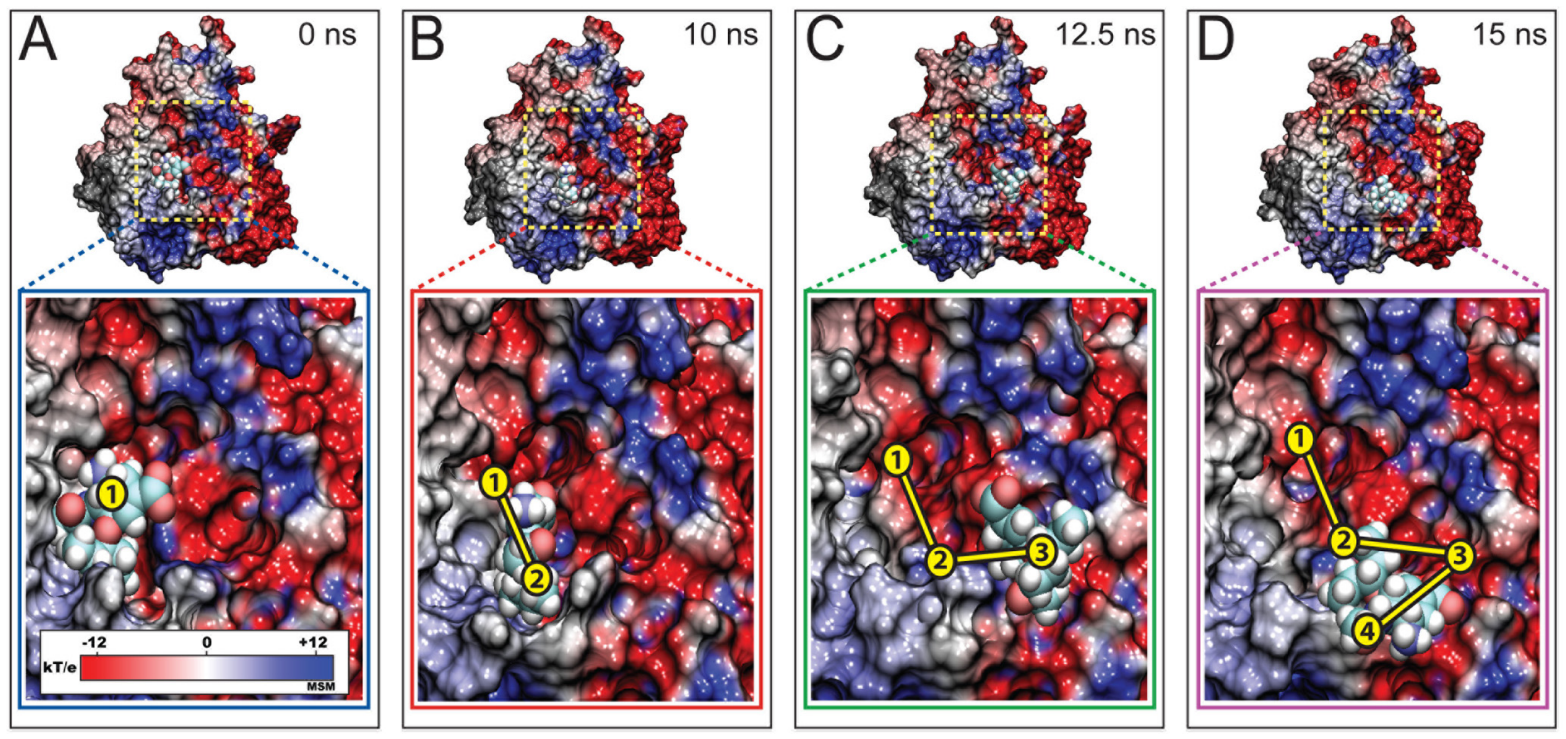
Forced unbinding of oseltamivir from H5N1 neuraminidase. Shown are timelapsed snapshots of oseltamivir along its exit pathway on the electrostatic surface of avian H5N1 neuraminidase during simulation *simSMD1*. At 0 ns (A), oseltamivir is stably bound within the SA binding pocket, as also seen in *simEQ1*. Application of force ruptures the stabilizing hydrogen bonds between H5N1 and oseltamivir (see also Figure 2), drawing the drug away from the SA binding site within 10 ns, as shown in B. Over the next 2.5 ns of pulling, oseltamivir follows the charged binding funnel (shown in C) until it is completely free of the protein binding pocket after 15 ns, as shown in D. Despite application of force directed straight out of the SA binding site, the drug follows a lateral unbinding path through the negatively charged funnel shown in Figure 1.

Despite application of force straight out of the binding pocket, oseltamivir surprisingly did not unbind along the direction of the force, following, instead, a lateral unbinding path. This path is characterized through strong interaction of the drug with the negatively charged column of residues identified in the electrostatic potential seen in Figure 1. The path taken by the drug is shown in Figure 3A–D, and in Video S1 (all videos are provided in Supplementary Materials). Tracing the relative position of oseltamivir and all its possible hydrogen bonding pairs with residues located along this pathway, it was recognized that nonspecific electrostatic attractions are the predominant interactions between drug and protein. The lateral pathway taken by the unbinding drug suggests strongly that it functions as a binding funnel which directs oseltamivir into the SA binding pocket.

**Figure 4:**
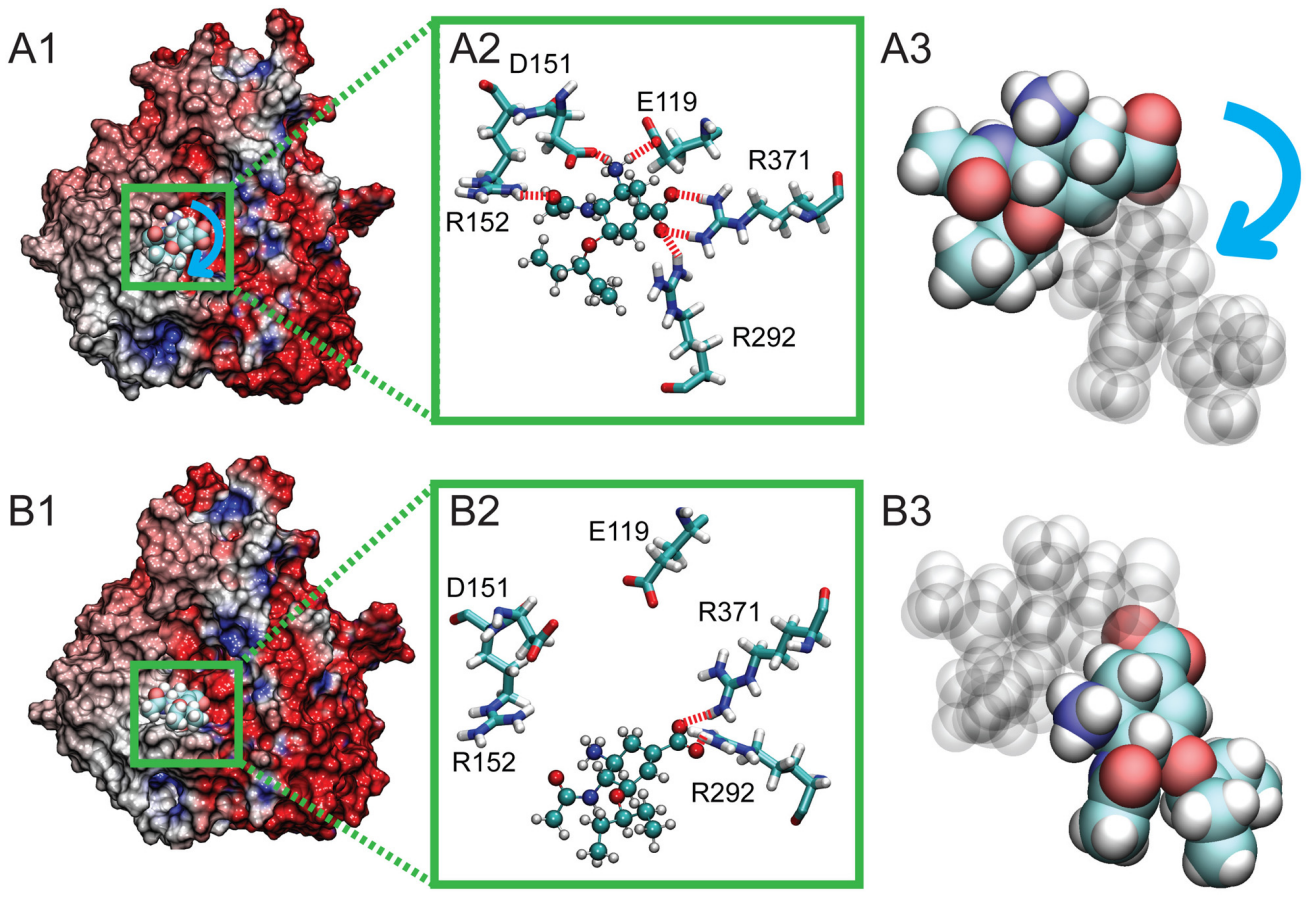
Rotated position of bound oseltamivir (arising at 7.5ns in *simSMD1*) in comparison with its stable equilibrium position (from *simEQ1*), shown with the electrostatic surface potential of the protein. In A1, the stable binding pose of oseltamivir is shown prior to application of force in *simSMD1*, with stable hydrogen bonds to E119, D151, R152, R292, and R371 (shown in A2). After 7.5 ns, however, oseltamivir adopted a new binding pose (shown in B1), where hydrogen bonds with E119, D151, and R152 are ruptured, leaving only stable interactions with R292 and R371 (shown in B2). The blue arrow in A3 denotes the orientation change during the drug's rotation in the binding pocket. The rotated bound oseltamivir shown in B1, B2, and B3 served as the starting point for simulations *simFEQ1–10*.

A key observation from *simSMD1* is that oseltamivir undergoes a rotation within the SA binding pocket before unbinding. This rotation, clearly discernible in Video S1, is the result of the rupture of hydrogen bonds between oseltamivir and residues E119, D151, and D152, while hydrogen bonds between oseltamivir's carboxyl functional group and residues R292 and R371 remain intact. The rotation, then, appears crucial for orienting oseltamivir into a position which permits it to more easily dissociate from the SA binding pocket. Oseltamivir in H5N1 is shown in its bound state before and after rotation in Figures 4A1–2 and 4B1–2, respectively. A comparison of the relative orientation of oseltamivir between the two states is provided in Figure 4A3 and 4B3, the latter showing the rotated state.

### Verifying the drug passage funnel through equilibrium simulations

SMD simulations are capable of capturing drug unbinding by accelerating the event through an applied force. It is desirable to verify SMD results through simulations without applied force. It was observed in *simSMD1* that following the transition to the rotated state, a much lower applied force is required to subsequently draw oseltamivir out of its binding pocket. This observation suggests that one may be able to probe the unbinding pathway without applied force, if oseltamivir is already in its rotated state. We performed, therefore, ten additional equilibrium simulations (*simFEQ1–10*) beginning with oseltamivir already in this state.

From simulations *simFEQ1–10*, two distinct outcomes were observed, namely the escape of oseltamivir from the SA binding pocket through favorable interactions with the charged binding funnel and a return of oseltamivir, not unexpectedly, to its pre-rotation bound state. Each simulation was carried out with sufficient duration to observe either outcome, with the exception of *simFEQ5*. In *simFEQ5*, oseltamivir, after following the binding funnel to escape the protein, actually rebound to the SA binding pocket through the same binding funnel, the dramatic return being captured in Video S6. A summary of observed outcomes from these simulations is shown in Table 2.

**Table 2:** Summary of *FEQ1–10* simulations starting from the rotated position of oseltamivir taken from *simSMD1* at 7.5 ns.

Name	Result	Time (ns)
simFEQ1	Drug escape via binding funnel	15
simFEQ2	Drug escape via binding funnel	10
simFEQ3	Drug escape via binding funnel	15
simFEQ4	Drug interaction with binding funnel but escape via 430-cavity	50
simFEQ5	Drug escape and rebinding into SA pocket via binding funnel	100
simFEQ6	Drug returned to unrotated bound position	10
simFEQ7	Drug returned to unrotated bound position	15
simFEQ8	Drug returned to unrotated bound position	50
simFEQ9	Drug returned to unrotated bound position	50
simFEQ10	Drug returned to unrotated bound position	50

In *simFEQ1–5*, oseltamivir successfully escaped the SA binding site, whereas in *simFEQ6–10*, oseltamivir returned to its stably bound unrotated state.

**Figure 5:**
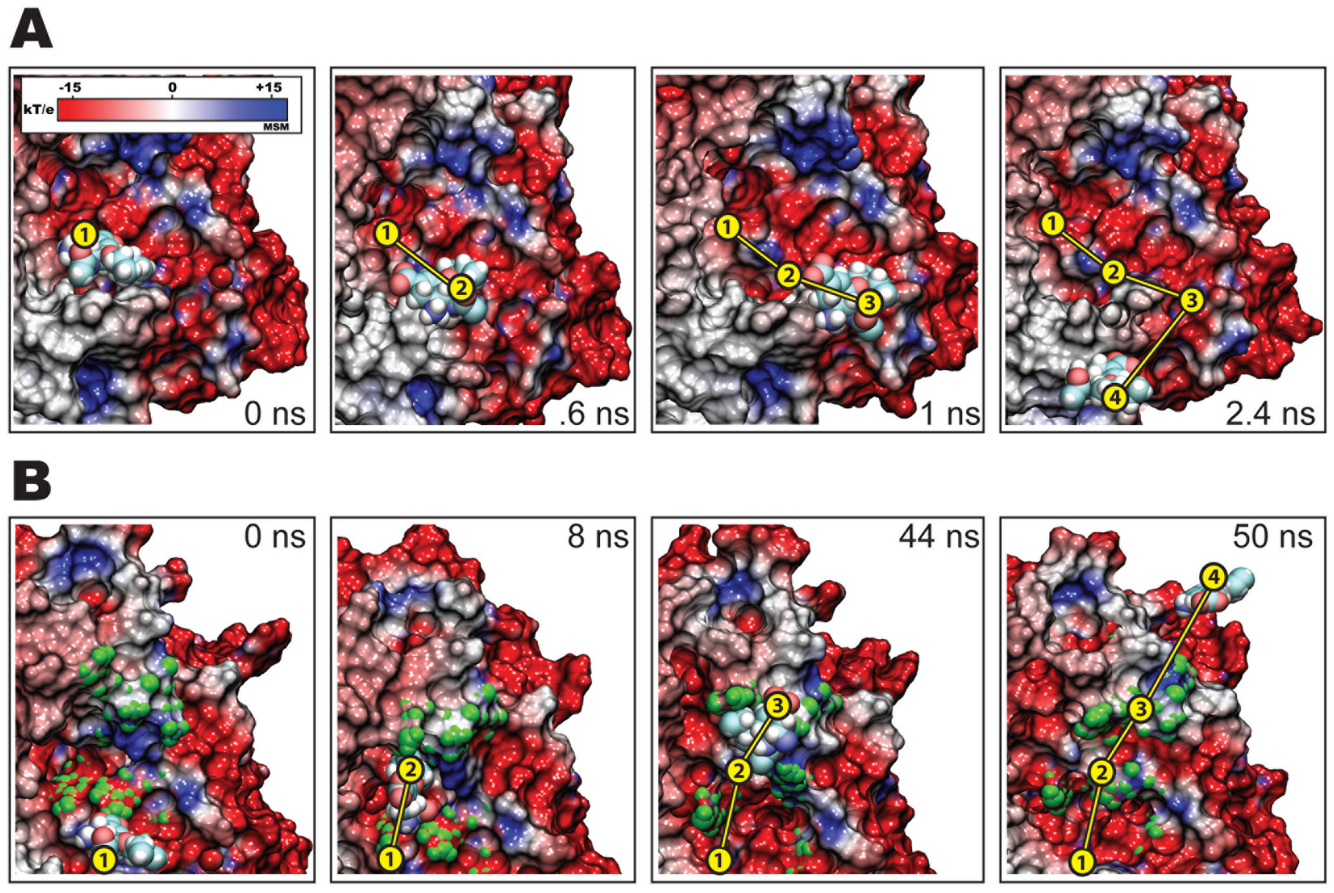
Escape of oseltamivir from H5N1 neuraminidase in equilibrium simulations from *simFEQ1* and *simFEQ4*. The initial state of the simulations is the rotated bound state of oseltamivir, illustrated in Figure 4. Shown in A) are snapshots from *simFEQ1*, showing the interaction of oseltamivir with H5N1's electrostatically charged binding funnel as the drug diffuses out of the neuraminidase SA binding pocket. Results were similar for *simFEQ2–3* and *simFEQ5* (see text and Table 2 for the specific timescales for the events), demonstrating a key role for this charged funnel in directing the drug into and out of the SA binding site. In snapshots from *simFEQ4*, (shown in B), oseltamivir briefly interacts with the binding funnel, but takes a different path out of the binding pocket, namely one interacting with residues from the so-called “430-cavity”, suggested in a prior study [15] to be a secondary SA binding site.

Oseltamivir freely, i.e., without external force applied, diffused out of the SA binding pocket by following the electrostatically charged binding funnel (described above) in five out of ten simulations (*simFEQ1–5*). In four cases (*simFEQ1–3, 5*) oseltamivir diffused along the full length of the binding funnel before separating from neuraminidase. Snapshots from a representative simulation (in this case *simFEQ1*) illustrating the trajectory that oseltamivir follows along our proposed binding funnel are shown in Figure 5A. This trajectory and those from *simFEQ2,3, and 5* can be inspected in Video S2, Video S3, Video S4, and Video S6. In *simFEQ4*, oseltamivir was observed to briefly interact with the binding funnel, but dissociated from neuraminidase through an alternate path, namely, through interaction with the “430-cavity” identified in earlier computational studies [15], [28]. This trajectory is shown in Video S5. Our simulation revealed that hydrogen bonds between oseltamivir's carboxylate group and the guanidino group of R430 appear to stabilize the transition of the drug along this alternate pathway. The 430-cavity is believed to function as a secondary binding site for SA, and our simulation (namely, *simFEQ4*) shows that it may (not surprisingly) also serve as a viable conduit for the binding/unbinding of oseltamivir. Snapshots from *simFEQ4* illustrating the trajectory of oseltamivir through the 430-cavity are shown in Figure 5B, with 430-cavity-specific residues colored in green.

**Figure 6:**
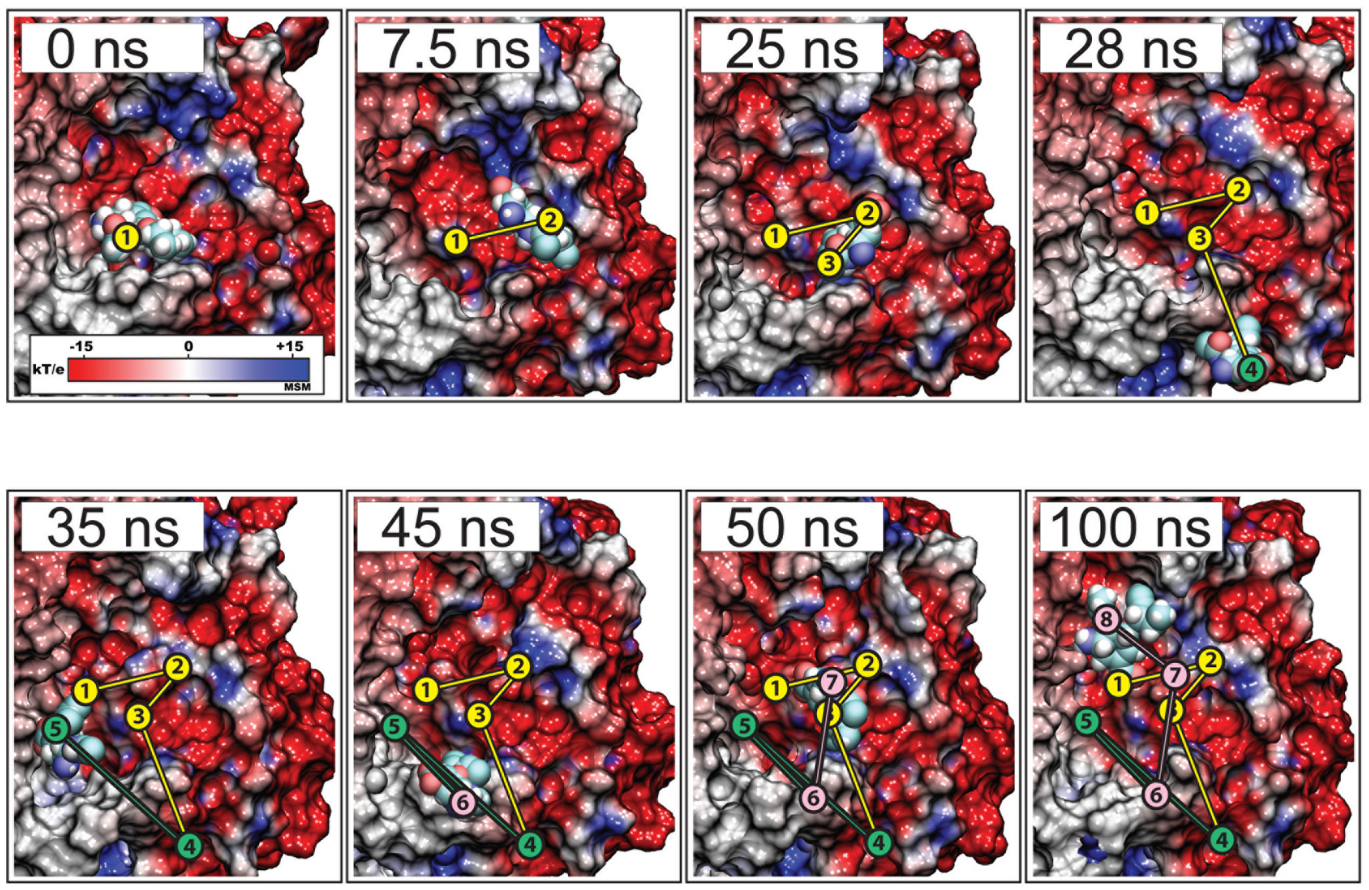
Escape and rebinding of oseltamivir through the electrostatic binding funnel in H5N1 neuraminidase during *simFEQ5*. Shown here are snapshots of *simFEQ5*, in which oseltamivir first diffuses out of the SA binding pocket through interaction with the electrostatic binding funnel (see Figure 1) similar to that seen in *simFEQ1–3* (see Figure 5A) within the first 25 ns of simulation. Between 28 and 35 ns, oseltamivir, now free from neuraminidase, approaches the periphery of the binding pocket away from the binding funnel, but is prohibited from entering due to electrostatic repulsion (45 ns). However, between 45 and 50 ns, oseltamivir reapproaches and rebinds with neuraminidase through the same, i.e., the electrostatic, binding funnel, adopting a stable position within the SA binding pocket through hydrogen bonds with Y406, R292, D151, E119, R118.

In *simFEQ1–4*, once oseltamivir separated from neuraminidase, the drug diffused into the surrounding solvent environment and away from the protein. However in *simFEQ5*, we observed not only a diffusion of oseltamivir through the charged binding funnel, but also the reentry of the drug through the same pathway after it had diffused already away from neuraminidase. Specifically, the sequence we observed in *simFEQ5* was: 1) between 0 to 25 ns, oseltamivir diffused out of neuraminidase's SA binding pocket by following the charged binding funnel, 2) between 25 and 35 ns, the drug unsuccessfully attempted to rebind from an unsuitable direction through hydrogen bond interactions with R152 in the so-called flexible 150-loop [29]; 3) between 35 and 45 ns, the drug again diffused away from neuraminidase; 4) between 45 and 50 ns, the drug approached the binding funnel again, drawing itself back into the funnel and binding at the SA binding pocket for 5) at least the next 50 ns (100ns total simulation time). Snapshots from these events are shown in Figure 6. Analysis of interactions of the newly rebound oseltamivir with binding pocket residues during the 50 to 100 ns interval revealed that the drug was stabilized by hydrogen bonds with Y406, R292, D151, E119, and R118, even though oseltamivir's pentyl group had not yet moved to its requisite hydrophobic pocket (I222-R224-A246-E276) [30]. The full *simFEQ5* trajectory is provided in Video S6, illustrating the strongest evidence observed thus far that the electrostatic funnel serves a crucial role in binding of oseltamivir. We note that while *simSMD1–3* and *simFEQ1–10* simulated the H5N1 systems, the results should also apply for the H1N1pdm systems which share a highly conserved drug active site and a very high overall sequence homology.

## Discussion

### Electrostatic binding funnel and drug resistance

**Figure 7:**
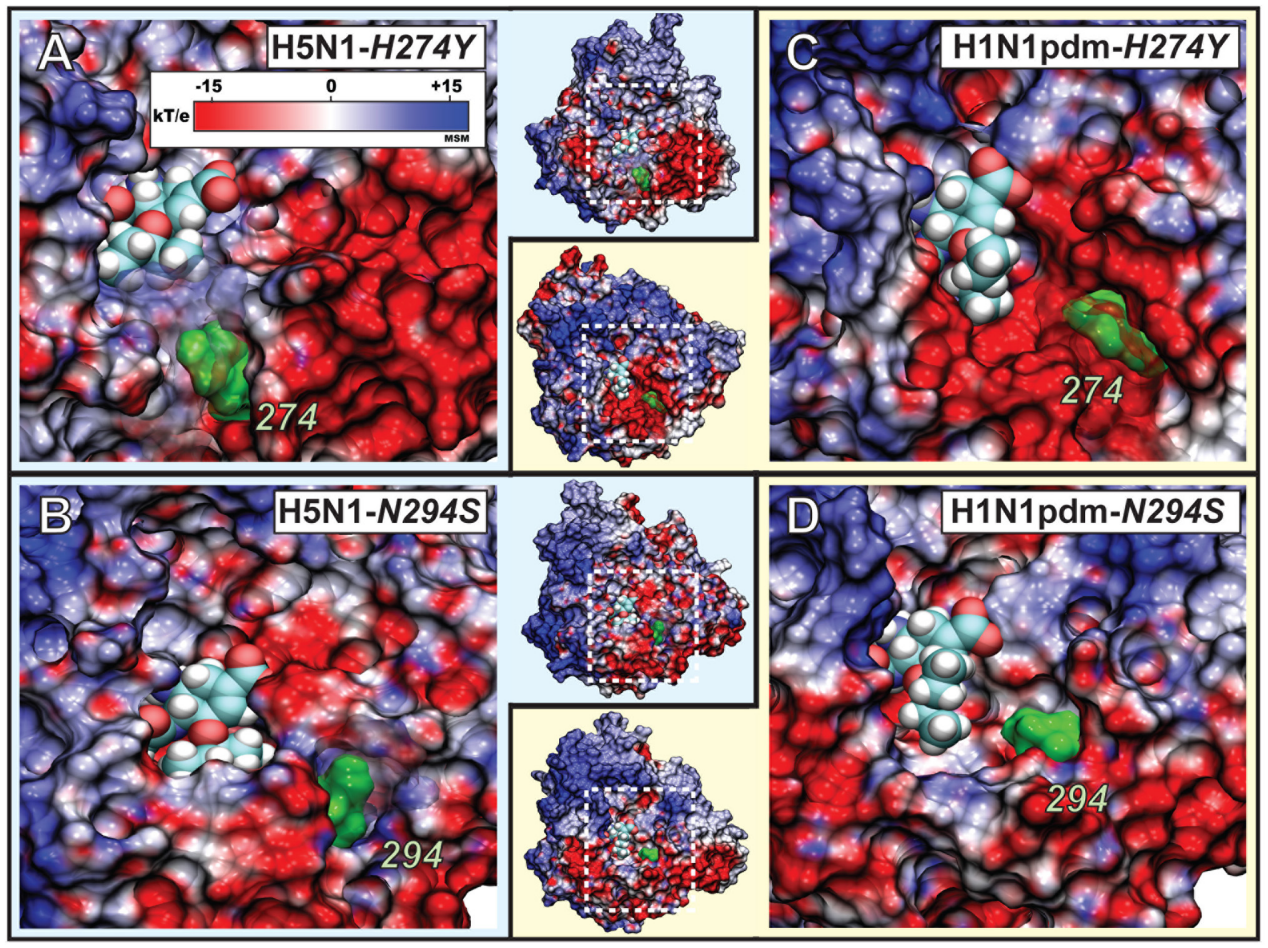
Possible effect of H274Y and N294S drug resistant mutations on the electrostatic binding funnel of H5N1 and H1N1pdm neuraminidases. Shown in A) and B) is oseltamivir bound to the H5N1 H274Y (*simEQ3*), and N294S (*simEQ5*) drug-resistant mutants, respectively. Shown in C) and D) is oseltamivir bound to H1N1pdm H274Y (*simEQ4*) and N294S (*simEQ6*) drug-resistant mutants, respectively. The outer columns show a close-up view of the binding pocket, highlighted as a subset of the entire protein shown in the central column. The positions of the mutant residues are shown in green for residue 274 and 294. Simulations *simEQ3–6* revealed the presence of a negatively charged funnel at the mouth of the binding pocket which may play a role in drug binding and mutation-induced resistance. Residues 274 and 294 are close to the residues involved in the binding funnel.

Our study has shed light on the important role of the electrostatic surface potentials in directing the diffusion of oseltamivir into the SA binding site of neuraminidase. The simulations yield strong evidence that the negatively charged funnel identified in this study serves as an unbinding pathway for oseltamivir in the H5N1 and, due to sequence and structural identity, also H1N1pdm wild type systems. The presence of a binding funnel raises an obvious question: would it be possible for the drug resistance mutations, in addition to their effects on destabilizing the hydrophobic packing of oseltamivir, to disrupt or otherwise alter this binding funnel? The conspicuous location of residue 294, which maps directly onto this negatively charged pathway, may play a key role in the N294S mutation for disrupting the proper guidance of the drug into its binding pocket. The H274Y mutation may also have a similar effect on drug binding, besides disrupting a hydrogen bond with R152. In all four simulations of the mutants (*simEQ3–6*), the positions of residues 274 (shown in Figures 7A and 7C) and 294 (shown in Figures 7B and 7D) lie directly on the charged binding funnel. An allosteric contribution due to the flexibility of the channel or that of the drug cannot be ruled out, i.e., the presence of the drug within the binding funnel may induce local conformational changes that bring the mutant residues into play, in which case electrostatic interactions may also be altered.

While active site interactions, including hydrogen bonds, hydrophobic packing, and solvent permeation, of oseltamivir resistance have been thoroughly studied [8],[10]–[12],[31] little is known at the atomic level about the kinetics of drug binding in mutants. The idea that drug resistant mutants actually disrupt entry of oseltamivir into the SA binding site of neuraminidase through disruption of an electrostatic binding funnel is in part supported by experiments which demonstrated altered drug binding kinetics in H5N1 H274Y and N294S mutants. Specifically, the reported association rate constants (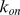) of oseltamivir with H5N1 neuraminidases were 2.52 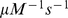 in WT, 0.24 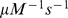 in H274Y and 1.1 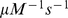 in N294S [12]. Even though the oseltamivir-resistant mutations were seen located in or adjacent to the funnel, additional study is still needed for a full understanding of whether the H274Y and N294S mutations weaken the binding of the drug. Our observations of an electrostatically active binding pathway for oseltamivir provides guidance for further investigations.

### Binding funnels on other neuraminidase subtypes

Our simulations were restricted to studying six subtypes of N1 neuraminidase; expanding the scope of investigation to encompass N2–9 neuraminidases may reveal similar electrostatic funnels as seen in H5N1 and H1N1pdm neuraminidases. While the N1 subtype neuraminidases are different from other subfamilies (N2–N9) [13], there is evidence that electrostatic interactions play also a key role in mediating ligand recognition in N2 [16] and in ligand binding in N9 [17]. Thus, even if the charge distribution pattern in the N2–N9 families turns out to differ significantly from that of N1, an approach similar to our study (or those of previous studies employing complementary methods to calculate electrostatic potentials) could shed light on mechanisms of drug binding or resistance mechanisms in these subtypes.

### Role of loop-150 and loop-430 in binding of oseltamivir

Previous studies [17], [29] suggest that two flexible loops (termed “150” and “430” due to their residue positions) play a role in guarding drug access to the SA binding pocket of N1 neuraminidases. A discussion on the positions of these loops relative to the binding funnel are provided in Text S4, and illustrated in Figure S7. Our simulations (*simEQ3* and *simEQ4*) reveal a loss of hydrogen bonds to loop 150 (namely between oseltamivir's acetyl group and R152) in the H274Y mutant systems. However, the disruption of endpoint interactions between oseltamivir and loop 150 do not appear to appreciably alter overall drug binding within the primary SA active site. This observation does not rule out what role loop 150 (and its associated D151 and R152 hydrogen bonds to oseltamivir) or loop 430 may play if the drug is occupying a secondary SA binding site (the “430 cavity”) suggested in [15], [16] and seen to play a role in simulation *simFEQ4*.

Electrostatic maps suggest a drug binding/unbinding pathway for oseltamivir along the charged binding funnel to the primary SA binding site, but *simFEQ4* suggests also a role of the “430-cavity” (via interaction between oseltamivir's carboxylate group and R430's guanidino group). Since our simulations, from which electrostatic maps were derived, modeled oseltamivir in its primary binding site, a secondary charged binding channel exit path may emerge when the drug occupies this secondary site which should be investigated further.

### Design against drug-resistant N1 influenza strains

The H274Y mutation induces drug-resistance to peramivir (a phase-III candidate), but neither H274Y nor N294S alter significantly the binding affinity for another antiviral drug, zanamivir [12], [32] or for sialic acid (SA), neuraminidase's natural substrate. The mechanism behind the efficacy of zanamivir against oseltamivir-resistant neuraminidase is not well understood. Examination of the crystal structure in mutants suggests that even in the case of the H274Y mutation, the zanamivir bound system is capable of maintaining a stabilizing hydrogen bond with residue 276 which is lost in the oseltamivir-bound mutant system [12]. Zanamivir's potency against oseltamivir-resistant strains also may be due to the high structural similarity it shares with SA. Specifically, zanamivir and SA share a hydrophilic glycerol group which is replaced by a hydrophobic pentyl group in oseltamivir and peramivir. Furthermore, there is evidence from prior studies [15], [16] that SA may follow a pathway into the neuraminidase active site which differs from that of oseltamivir; zanamivir may follow the same pathway as SA, and, hence, alteration of the oseltamivir binding funnel may not affect zanamivir, despite zanamivir's increased polarization relative to oseltamivir. In contrast, peramivir's ineffectiveness in the case of the H274Y mutant may be due to a change in hydrophobic packing of its pentyl group as witnessed in our simulations for oseltamivir. Recent efforts to derive new drugs that are effective against neuraminidase have also suggested inhibitors with remarkably different scaffolds than oseltamivir, zanamivir, and peramivir [33], [34]. Therefore, rational drug design for neuraminidase inhibitors will certainly benefit from studies that focus on binding kinetics behind selective drug resistance and also consider electrostatic steering [15]–[18].

### Conclusion

Our simulations of wild type and mutant H5N1 and H1N1pdm neuraminidase systems bound to oseltamivir suggest that while the H274Y and N294S mutations appear to mainly disrupt the hydrophobic packing of oseltamivir's pentyl sidegroup, but not necessarily its conserved hydrogen network, drug-resistance may also arise from disruption of the binding process, i.e., from altered kinetics. Our simulations reveal a charged pathway in both H5N1 and H1N1pdm neuraminidase, that functions as a binding conduit for oseltamivir in which drug passage is controlled primarily by electrostatic attraction between drug and protein. The insight gained should assist in the rational design of neuraminidase inhibitors that exploit this binding pathway, but also avoid drug resistance.

## Methods

### Molecular model of H1N1pdm neuraminidase

The amino acid sequence of H1N1pdm neuraminidase was obtained from Genbank Locus ID CY041156, and that of H5N1 neuraminidase from Protein Data Bank entry 2HU4 [13]. A sequence alignment performed using Multiseq in VMD [35] showed that H1N1pdm has the highest percent of sequence identity (91.47%) with H5N1 among all neuraminidases with high resolution structural data. At the drug binding pocket, the notable difference between H5N1 and H1N1pdm neuraminidases is the replacement of Y347 by N347. Therefore, a homology model of H1N1pdm was built using H5N1 as the starting point and by mutating corresponding residues to match the wild type H1N1pdm.

### Preparation of starting structures

The coordinates for H5N1 neuraminidase bound with oseltamivir (Tamiflu) was taken from a monomer of Protein Data Bank (PDB) structure 2HU4 (tetramer) [13], while those of mutants H274Y and N294S were taken from structures 3CL0 (monomer) and 3CL2 (monomer), respectively [12]. Even though the tetrameric form of neuraminidase has been employed in previous simulations [15], [17], its monomer contains a functionally complete active site and has been shown to yield satisfactory results in prior studies [8], [18], [31]. Therefore to conserve computational resources, only single monomers were used in our simulations. The position for oseltamivir bound to H1N1pdm was adopted from its corresponding location in H5N1, as the two proteins' binding pockets differ only by residue 347, located on a loop at the periphery of the active site. Oseltamivir-mutant complexes of H1N1pdm were built by mutating H274Y and N294S of the H1N1pdm wild type model. An representative simulated system is illustrated in Figure S8.

### Simulated systems

The simulations carried out are listed in Table 1. Six systems were modeled and simulated, namely oseltamivir bound H5N1, and H1N1pdm wild type, and H274Y as well as N294S mutants. The first six simulations involved equilibration of each oseltamivir bound neuraminidase structure (*simEQ1–6*). In *simSMD1*, steered molecular dynamics (SMD) simulations were used to remove oseltamivir from its stable binding site in H5N1 neuraminidase. In *simFEQ1–10*, equilibration simulations used a starting point generated from *simSMD1* in which oseltamivir had undergone a rotation which partially displaced the drug from the binding site. In total, 680 ns of simulation were carried out for a system size of about 35,000 atoms.

Crystallographically resolved water molecules and a structurally relevant calcium ion near the native binding site for sialic acid (SA) were retained and modeled in all systems simulated. The protein complexes were then solvated in a TIP3P water box [36] and ionized by NaCl (0.152M) to mimic physiological conditions. The solvated H1N1pdm system with bound oseltamivir and active site calcium ion is shown in Figure S7A, a schematic view of the buried drug in the SA binding site is shown in Figure S7B.

### Parameter generation for oseltamivir

Simulation parameters for oseltamivir were developed under the CHARMM force field parameter scheme, to complement the CHARMM31 force field for proteins with CMAP correction [37], [38]. Parameters for ligands were prepared using Paratool [39] in VMD [35]. Structure optimization and frequency calculations were performed at the HF/6-31G* level of Gaussian03 [40] and subsequently imported into Paratool. The quantum mechanics frequency calculation of the optimized geometry produced Hessian matrices which were transformed into the set of internal coordinates describing the drug bonded interactions. Atom types and charges already described in the existing CHARMM force field were assigned using the existing parameters. The atomic charges in oseltamivir's six member ring were newly parameterized by dividing the ring into several small fragments, and recalculated based on the total charge of each fragment. Fragments not explicitly defined in the CHARMM force field were modeled using analogs in the CHARMM force field extensions which fit closely to the fragment being parameterized. The dihedral angle potentials, which render the respective torsions highly rigid due to electron delocalization, were generated from the Hessian matrices. The drug's parameter and topology files are included in Supplemental Materials, as Protocol S1 and Protocol S2, respectively.

### Molecular dynamics simulations

All simulations were performed using NAMD 2.7b2 [41] and the CHARMM31 force field with CMAP correction [37], [38]. The ionized systems were minimized for 10,000 integration steps and equilibrated for 20 ns with 1 fs time stepping. Following this, a 20 ns unconstrained equilibration was performed for subsequent trajectory analysis, with frames stored each picosecond. Constant temperature (T = 300 K) was enforced using Langevin dynamics with a damping coefficient of 1 ps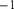. Constant pressure (p = 1 atm) was enforced through the Nosé-Hoover Langevin piston method with a decay period of 100 fs and a damping time constant of 50 fs. Van der Waals interaction cutoff distances were set at 12 Å, (smooth switching function beginning at 10 Å) and long-range electrostatic forces were computed using the particle-mesh Ewald (PME) with a grid size of less than 1 Å.

SMD simulations [42]–[47] fixed the center of mass of neuraminidase 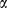-carbons and applied a force to the center of mass of oseltamivir, along a vector connecting the two center of masses. In *simSMD1–3*, a constant velocity protocol was employed, with a pulling velocity of 0.5 Å/ns, 0.10 Å/ns, and 0.25 Å/ns, respectively. For the SMD spring constant [22], [48], we chose 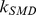 = 3*k* 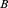 *T*/Å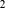 which corresponds to an RMSD value of 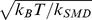 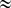
0.6 Å.

### Analysis

Trajectory frames were saved every 1000 integration timesteps (every picosecond). Analysis included the calculation of an averaged electrostatic potential field over all frames of a trajectory using RMSD-aligned structures. Maps of the electrostatic potential field were calculated on a three-dimensional lattice. The long-range contributions to the electrostatics were calculated employing the multilevel summation method (MSM), which uses nested interpolation of the smoothed pairwise interaction potential, with computational work that scales linearly with the size of the system [49]. The calculation was performed using the molecular visualization program VMD [35] that provides a GPU-accelerated version of MSM to produce the electrostatic potential map [21]. The GPU acceleration of MSM provided a significant speedup over conventional electrostatic summation methods such as the Adaptive Poisson Boltzman Solver (APBS) [50]. In fact, we achieved a processing time of 0.2 s per frame, versus 180 s per frame (on a conventional CPU) using APBS for a 35,000 atom system, which corresponds to a speedup factor of about 900. The use of GPU acceleration permitted averaging the electrostatic potential field over all frames of our simulation trajectories.

The root mean square deviation (RMSD) for the position of atoms within the simulation systems were used to access protein stability and state of equilibration. The RMSD calculations took into account a total frame alignment for the 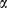-carbons of either drug only or protein only, depending on the value reported (Figure S1 and Figure S2A). In accessing the stability of oseltamivir, we also aligned our coordinates against active site residues only (117–119, 133–138, 146–152, 156, 179, 180, 196–200, 223–228, 243–247, 277, 278, 293, 295, 344–347, 368, 401, 402, and 426–441, taken from [15]) for an additional drug-only RMSD calculation (Figure S3). For hydrogen bond analysis, a distance and angle cutoff of 3.5 Å and 60 degrees were employed, respectively. The change to the amount of solvent accessible surface area was used to assess alterations of hydrophobic packing interactions. The presence of salt bridges was assessed by taking a nitrogen-oxygen cutoff distance of 3.2 Å between charged residue side chains. Any close contacts that fell outside of the bound of molecular bonds or electrostatic (hydrogen bond or salt bridge) interactions were then examined frame-by-frame to distinguish between nonspecific surface or charged contacts.

## Supporting Information

Figure S1 Root mean squared deviation (RMSD) of WT and mutant avian H5N1 and swine H1N1pdm neuraminidases across six 20ns simulations (simEQ1 to simEQ6). The values reflect the equilibration of each of the neuraminidase systems.
(1.85 MB TIF)

Figure S2 Root mean squared deviation (RMSD) of oseltamivir within the sialic acid (SA) binding pocket of WT and mutant avian H5N1 and swine H1N1pdm, respectively, across six 20 ns simulations (simEQ1 to simEQ6 aligned by drug position). The values show that the positions of the drug remain fairly constant with minimal deviation within the binding pocket, thereby permitting the characterization of the specific drug-protein interactions responsible for binding oseltamivir to the active site of neuraminidase N1 subtypes.
(1.77 MB TIF)

Figure S3 Root mean squared deviation (RMSD) of oseltamivir within the sialic acid (SA) binding pocket of WT and mutant avian H5N1 and swine H1N1pdm, respectively, across six 40ns simulations (simEQ1 to simEQ6 aligned by active site residues). The relative motion of oseltamivir in the mutant systems can be attributed to a rotation of its pentyl group. However, over the entire simulation trajectory the drug remained bound to the neuraminidase active site.
(3.04 MB TIF)

Figure S4 Network and occupancy of hydrogen bonds stabilizing oseltamivir in the SA binding pocket of wild type and drug-resistant mutant avian H5N1 neuraminidases, in simEQ1–3. A) Histograms of hydrogen-bond occupancies for interactions between oseltamivir and residues E119, D151, R152, R292, Y347, and R371 across each simulation run. B) through D) Schematic views depicting the orientation of protein sidechains which form protein-drug hydrogen bonds. Hydrogen bonds in all three simulations were conserved for residues E119, D151, R292, and R371. The H274Y mutation was observed to disrupt hydrogen bonding to R152. Despite the increased interaction with Y347 in the case of the N294S mutant, the hydrogen bonds between oseltamivir and Y347 were not stable in any of the simulated systems.
(1.48 MB TIF)

Figure S5 Network and occupancy of hydrogen bonds stabilizing oseltamivir in the SA binding pocket of wild type and drug-resistant mutant avian H1N1pdm neuraminidases, in simEQ4–6. A) Histograms of hydrogen-bond occupancies for interactions between oseltamivir and residues E119, D151, R152, R292, N347, and R371 across each simulation run. B) through D) Schematic views depicting the orientation of protein sidechains which form protein-drug hydrogen bonds. Hydrogen bonds in all three simulations were conserved for residues E119, D151, R292, and R371. The H274Y mutation was observed to disrupt hydrogen bonding to R152. Interestingly, residue 347, which distinguishes the binding pocket of H1N1pdm from H5N1, makes no contribution to the drug-protein hydrogen-bond network in the case of H1N1pdm.
(1.45 MB TIF)

Figure S6 Solvent accessible surface area of oseltamivir's pentyl group (PG-SASA) in H5N1 and H1N1pdm WT and mutant simulations.
(2.08 MB TIF)

Figure S7 The position of flexible loops 150 and 430 relative to the charged binding funnel of H5N1 neuraminidase.
(2.31 MB TIF)

Figure S8 Drug bound systems simulated. Shown here is a representative example of H1N1pdm bound to oseltamivir. In A), the system is shown in the solvation box and with oseltamivir and the active site calcium ion. In B), oseltamivir is shown buried in the SA binding pocket of H1N1pdm, the latter rendered in surface view.
(1.67 MB TIF)

Protocol S1 CHARMM parameter file for oselvamivir.
(0.01 MB TXT)

Protocol S2 CHARMM topology file for oseltamivir.
(0.02 MB TXT)

Text S1 Supplementary material text S1.
(0.02 MB PDF)

Text S2 Supplementary material text S2.
(0.04 MB PDF)

Text S3 Supplementary material text S3.
(0.03 MB PDF)

Text S4 Supplementary information Text S4.
(0.02 MB PDF)

Video S1 Trajectory from simSMD1, where a force is applied to oseltamivir perpendicular to the plane of the SA binding pocket. Despite the direction of force, oseltamivir interacts with and follows the charged electrostatic pathway identified and discussed in Figure 1, with snapshots shown in Figure 3. Format: AVI.
(7.30 MB AVI)

Video S2 Trajectory from simSMD1, where a force is applied to oseltamivir perpendicular to the plane of the SA binding pocket. Despite the direction of force, oseltamivir interacts with and follows the charged electrostatic pathway identified and discussed in Figure 1, with snapshots shown in Figure 3. Format: MOV.
(7.29 MB MOV)

Video S3 Depicts the trajectory from simFEQ1, showing the spontaneous diffusion of oseltamivir out of the neuraminidase binding pocket via interaction with the electrostatic binding funnel (see Figure 3 for snapshots from simFEQ1). Format: AVI.
(4.16 MB AVI)

Video S4 Depicts the trajectory from simFEQ1, showing the spontaneous diffusion of oseltamivir out of the neuraminidase binding pocket via interaction with the electrostatic binding funnel (see Figure 3 for snapshots from simFEQ1). Format: MOV.
(4.15 MB MOV)

Video S5 Depicts the trajectory from simFEQ2, showing the spontaneous diffusion of oseltamivir out of the neuraminidase binding pocket via interaction with the electrostatic binding funnel (see Figure 3 for snapshots from simFEQ1). Format: AVI.
(4.17 MB AVI)

Video S6 Depicts the trajectory from simFEQ2, showing the spontaneous diffusion of oseltamivir out of the neuraminidase binding pocket via interaction with the electrostatic binding funnel (see Figure 3 for snapshots from simFEQ1). Format: MOV.
(4.17 MB MOV)

Video S7 Depicts the trajectory from simFEQ3, showing the spontaneous diffusion of oseltamivir out of the neuraminidase binding pocket via interaction with the electrostatic binding funnel (see Figure 3 for snapshots from simFEQ1). Format: AVI.
(4.14 MB AVI)

Video S8 Depicts the trajectory from simFEQ3, showing the spontaneous diffusion of oseltamivir out of the neuraminidase binding pocket via interaction with the electrostatic binding funnel (see Figure 3 for snapshots from simFEQ1). Format: MOV.
(4.13 MB MOV)

Video S9 Depicts the trajectory from simFEQ4, where oseltamivir briefly interacts with the electrostatic binding funnel before diffusing out of the neuraminidase binding pocket via an alternate pathway in the region of the “430-loop” shown in green in Figure 5. Format: AVI.
(4.09 MB AVI)

Video S10 Depicts the trajectory from simFEQ4, where oseltamivir briefly interacts with the electrostatic binding funnel before diffusing out of the neuraminidase binding pocket via an alternate pathway in the region of the “430-loop” shown in green in Figure 5. Format: MOV.
(4.09 MB MOV)

Video S11 Depicts the trajectory from simFEQ5, where oseltamivir diffuses out of the neuraminidase active site via interaction with the electrostatic binding funnel, fails to enter the active site at a different location on the periphery of the binding pocket due to electrostatic repulsion, then rebinds stably to neuramindase through the electrostatic binding funnel. See Figure 6 for snapshots. Format: AVI.
(8.47 MB AVI)

Video S12 Depicts the trajectory from simFEQ5, where oseltamivir diffuses out of the neuraminidase active site via interaction with the electrostatic binding funnel, fails to enter the active site at a different location on the periphery of the binding pocket due to electrostatic repulsion, then rebinds stably to neuramindase through the electrostatic binding funnel. See Figure 6 for snapshots. Format: MOV.
(7.74 MB MOV)
