# Supplementary figures and images for: Molecular Dynamics Simulations Suggest that Electrostatic Funnel Directs Binding of Tamiflu to Influenza N1 Neuraminidases
Source: PLoS Comput Biol. 2010 Sep 23;6(9):e1000939. doi: 10.1371/journal.pcbi.1000939 (PMC2944783; doi:10.1371/journal.pcbi.1000939)

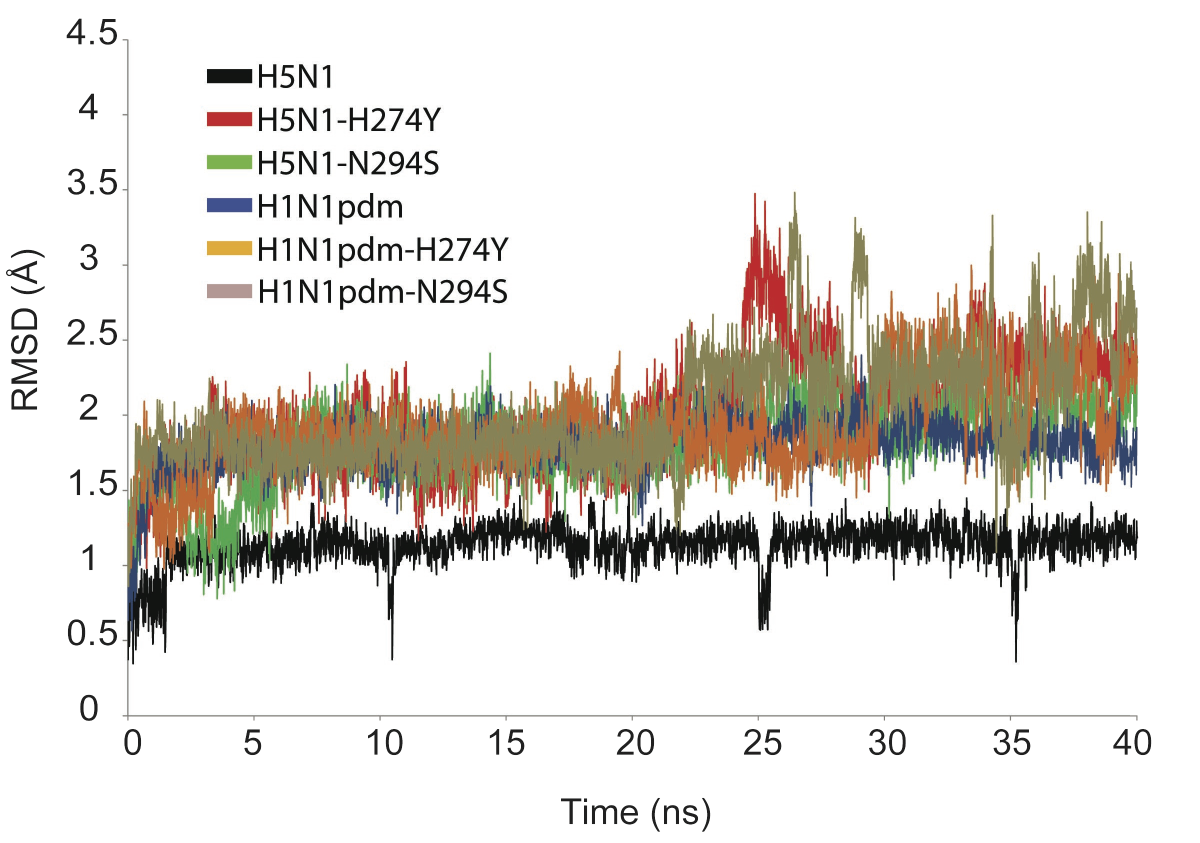

Supplement: Figure S3 — Root mean squared deviation (RMSD) of oseltamivir within the sialic acid (SA) binding pocket of WT and mutant avian H5N1 and swine H1N1pdm, respectively, across six 40ns simulations (simEQ1 to simEQ6 aligned by active site residues). The relative motion of oseltamivir in the mutant systems can be attributed to a rotation of its pentyl group. However, over the entire simulation trajectory the drug remained bound to the neuraminidase active site. (3.04 MB TIF) [file pcbi.1000939.s003.tif]
