# Supplementary material for: Molecular Dynamics Simulations Suggest that Electrostatic Funnel Directs Binding of Tamiflu to Influenza N1 Neuraminidases
Source: PLoS Comput Biol. 2010 Sep 23;6(9):e1000939. doi: 10.1371/journal.pcbi.1000939 (PMC2944783; doi:10.1371/journal.pcbi.1000939)
Supplement: Text S1 — Supplementary material text S1. (0.02 MB PDF) [file pcbi.1000939.s011.pdf]

Supplementary Text S1

Oseltamivir bound stably to all H5N1 and H1N1pdm wild type and mutants.

The protein root mean squared deviation (RMSD) (Figure S1) of six simulated systems demonstrate that the simulated systems were stable within the timescale simulated. The RMSD of the drug plotted in Figures S2 and S3 over each simulation trajectory, shows that within the first 20 ns equilibrium simulations, the drug bound strongly to wildtype proteins. In mutant systems, drug RMSDs increased after 20ns of simulations. Figure S3, which plots the stability of the drug relative to the active site residues over the entire 40 ns trajectory, reveals movement of the drug that can be traced to fluctuations of its pentyl group (normally bound within a hydrophobic pocket of neuraminidase associated with residues 274 and 294) in the case of H274Y and N294S mutants. These observations support predictions derived from experiments that suggested a role the H274Y and N294S mutations played in disrupting the packing stability of oseltamivir's pentyl group [1]. Drug RMSDs were seen to vary in H274Y mutants to a higher degree than that in N294S systems, suggesting that H274Y mutation causes a larger disruption of the hydrophobic pocket stabilizing oseltamivir's pentyl group. However, all other critical drug-protein interactions, apart from those with oseltamivir's pentyl group, were observed to be fairly constant with deviations being smallest within the binding pocket; oseltamivir was observed after 40 ns of simulation to remain within the SA active site of all six neuraminidase systems simulated. To investigate if the H274Y and N294S mutations also rupture the hydrogen bonds responsible for stabilizing oseltamivir within the neuraminidase active site, we measured hydrogen bond formation between drug and all wildtype proteins as well as mutants.

References

1. Collins PJ, Haire LF, Lin YP, Liu J, Russell RJ, et al. (2008) Crystal structures of oseltamivir-resistant influenza virus neuraminidase mutants. Nature 453: 1258-1261.
