# Supplementary material for: Molecular Dynamics Simulations Suggest that Electrostatic Funnel Directs Binding of Tamiflu to Influenza N1 Neuraminidases
Source: PLoS Comput Biol. 2010 Sep 23;6(9):e1000939. doi: 10.1371/journal.pcbi.1000939 (PMC2944783; doi:10.1371/journal.pcbi.1000939)
Supplement: Text S2 — Supplementary material text S2. (0.04 MB PDF) [file pcbi.1000939.s012.pdf]

Text S2: Hydrogen bond networks connecting oseltamivir with H5N1 and H1N1pdm neuraminidases

In both wildtype (WT) and mutant simulations, hydrogen bonds between oseltamivir and binding site residues were well conserved, including E119, D151, R292, and R371. Specifically, R292 and R371 were observed to form a hydrogen bond with oseltamivir's carboxylate moiety, and E119 and D151 with oseltamivir's amino group. The H274Y mutation, however, appeared to disrupt the hydrogen bonding of oseltamivir's acetyl group with R152, an interaction which was seen in the wildtype and N294S systems both in *simEQ5* and *simEQ6*. The disruption of the R152 hydrogen bond in the H274Y mutant systems may be attributed to a rotation of oseltamivir's pentyl group due to disfavorable hydrophobic packing, as discussed below. To the best of our knowledge, the disruption of oseltamivir's R152 interaction in the H274Y mutant has not been reported in previous studies on the avian H5N1 H274Y mutant [1–3]. Results from our respective calculations are presented in Figures S4-A and S5-A, which show histograms of hydrogen bond formation frequency, with schematic views of the specific residues involved in hydrogen bonding in Figures S4-B to D for *simEQ1*, *simEQ3*, and *simEQ5*, respectively, and in Figures S5-B to D for *simEQ2*, *simEQ4*, and *simEQ6*, respectively.

Prior analyses of crystallographic data alone suggested that a hydrogen bond between Y347 and oseltamivir's carboxyl group, found in the wildtype structure, is lost in the case of the N294S mutant [3], suggesting one possible mechanism of drug resistance. Our simulations produce a dynamic picture of molecular interactions at great resolution that complements the static crystal structure, but do not reveal the presence of a stable hydrogen bond between drug and Y347, not even in the wildtype systems. Prior computational studies of an H5N1+oseltamivir system using the AMBER force field also failed to show evidence that the Y347 hydrogen bond is stable [4]. While it is well known that choice of force fields can introduce a bias for protein stability [5], the fact that the Y347 hydrogen bond fails to stay intact for either CHARMM and AMBER force field, supports our conclusion. In our simulations oseltamivir's carboxyl group was observed to primarily form hydrogen bonds with R292 and R371, having little involvement with Y347. In fact, residue Y347 undergoes rotation to interact strongly with residue W295. Therefore, the suggestion from previous studies on the H5N1 N294S mutant [2, 3], that the N294S mutation in case of H5N1 actually destabilizes hydrogen bonding between oseltamivir and Y347 to induce drug resistance, is not supported by our simulations.

The notable difference between H5N1 and H1N1pdm neuraminidases is the replacement of Y347 by N347 in the drug binding pocket. No conserved drug-protein hydrogen bond was observed for N347 in any of the three H1N1pdm simulations. Given the transient nature of even the N294S mutant induced hydrogen bond involving residue 347 in the case of H5N1, and the lack of interaction with residue 347 in any of the other simulations, it is highly unlikely that the single residue change (Y347 to N347), between the H5N1 and H1N1pdm strains, significantly alters the drug-protein stability in regard to the hydrogen bond network involved.

References

1. Malaisree M, Rungrotmongkol T, Nunthaboot N, Aruksakunwong O, Intharathep P, et al. (2009) Source of oseltamivir resistance in avian influenza H5N1 virus with the h274y mutation. Amino Acid 37: 725-732.

2. Nick XW, Zheng JJ (2009) Computational studies of H5N1 influenza virus resistance to oseltamivir. Prot Sci 18: 707-715.

3. Collins PJ, Haire LF, Lin YP, Liu J, Russell RJ, et al. (2008) Crystal structures of oseltamivir-resistant influenza virus neuraminidase mutants. Nature 453: 1258-1261.

4. Malaisree M, Rungrotmongkol T, Decha P, Intharathep P, Aruksakunwong O, et al. (2008) Understanding of known drug-target interactions in the catalytic pocket of neuraminidase subtype N1. Proteins: Struct, Func, Bioinf 71: 1908-1918.

5. Freddolino PL, Park S, Roux B, Schulten K (2009) Force field bias in protein folding simulations. Biophys J 96: 3772-3780.

KEY: FRED2009
ANNOTATION:
