# Supplementary material for: Molecular Dynamics Simulations Suggest that Electrostatic Funnel Directs Binding of Tamiflu to Influenza N1 Neuraminidases
Source: PLoS Comput Biol. 2010 Sep 23;6(9):e1000939. doi: 10.1371/journal.pcbi.1000939 (PMC2944783; doi:10.1371/journal.pcbi.1000939)
Supplement: Text S3 — Supplementary material text S3. (0.03 MB PDF) [file pcbi.1000939.s013.pdf]

Text S3: H274Y mutation disrupts hydrophobic packing of oseltamivir's pentyl group in both H5N1 and H1N1pdm neuraminidase

Beside disrupting the drug-protein hydrogen-bonding network, the N274Y mutation may induce drug resistance by disrupting the hydrophobic packing of the drug into the protein binding pocket. Through inspection of the static crystal structures of the H274Y and N294S mutants of H5N1, it has been suggested that the mutations disrupt favorable hydrophobic packing interactions necessary for strong binding of oseltamivir [1]. In our simulations of WT H5H1 and H1N1pdm, the packing of oseltamivir's pentyl moiety tends to favor close association with residues I222, R224, A246, and E276. To test the effect of mutations H274Y and N294S on hydrophobic interactions of oseltamivir's pentyl group with the proteins, we monitored the solvent accessible surface area (SASA) of oseltamivir's pentyl group for all simulation trajectories. While there was no significant change to the pentyl group SASA (henceforth referred to as PG-SASA) in the wildtype and N294S mutant for either H5N1 or H1N1pdm neuraminidases, an outward rotation of the pentyl group was observed for H274Y, discernable in oseltamivir's binding pose and evident in a notably higher calculated PG-SASA. The PG-SASAs for all simulated systems are shown in Figure S6, with inset images of oseltamivir's binding pose in simH1N1pdm (Figure S5A) and simH1N1pdm-H274Y (Figure S6B), illustrating the rotation of the pentyl group towards the open mouth of the binding pocket.

Previously published MD simulations performed over relatively short time scales (3 to 6 ns) have suggested two possible mechanisms: 1) that the H247Y mutation reduces the size of the hydrophobic pocket within the SA binding pocket near oseltamivir's pentyl moiety [2] and 2) that the H274Y mutation breaks a critical salt bridge between E276 and R224 to disrupt drug binding [3]. Our longer (40ns) simulations were able to corroborate mechanism 1 (as shown by the increase in PG-SASA in the case of the H274Y mutant simulations), but not mechanism 2. In fact, in all six of our simulations, E276 maintains stable charge-charge interactions (salt bridging) with R224 despite displacement of the drug from the protein I222-R224-A246-E276 pocket in the case of H274Y mutants. This drug displacement increases water penetration into the pocket (Figure S6). Our simulations support therefore predictions from earlier studies of a possible mechanism for the H274Y mutation-induced drug resistance through water infiltration and destabilization of favorable drug packing [1].

References

1. Collins PJ, Haire LF, Lin YP, Liu J, Russell RJ, et al. (2008) Crystal structures of oseltamivir-resistant influenza virus neuraminidase mutants. Nature 453: 1258-1261.

2. Malaisree M, Rungrotmongkol T, Nunthaboot N, Aruksakunwong O, Intharathep P, et al. (2009) Source of oseltamivir resistance in avian influenza H5N1 virus with the h274y mutation. Amino Acid 37: 725-732.

3. Nick XW, Zheng JJ (2009) Computational studies of H5N1 influenza virus resistance to oseltamivir. Prot Sci 18: 707-715.
