# Supplementary material for: Molecular Dynamics Simulations Suggest that Electrostatic Funnel Directs Binding of Tamiflu to Influenza N1 Neuraminidases
Source: PLoS Comput Biol. 2010 Sep 23;6(9):e1000939. doi: 10.1371/journal.pcbi.1000939 (PMC2944783; doi:10.1371/journal.pcbi.1000939)
Supplement: Text S4 — Supplementary information Text S4. (0.02 MB PDF) [file pcbi.1000939.s014.pdf]

Text S4: Orientation of loops 150 and 430 in relation to the charged binding funnel.

The electrostatic potential of N1 neuraminidase (as discussed in the main text) revealed a novel charged binding funnel which may direct the passage of oseltamivir. In previous studies, two flexible loops (150 and 430) at the periphery of the binding pocket appeared to play an important role in ligand binding [1,2]. The positions of these loops, though, are located sufficiently distant from the novel charged binding funnel such that they quite apparently do not function as gating elements for this particular pathway. This is illustrated in Figure S7.

References

1. Amaro RE, Minh DDL, Cheng LS, William M Lindstrom JAJO, Lin JH, et al. (2007) Remarkable loop flexibility in avian influenza N1 and its implications for antiviral drug design. J Am Chem Soc 129: 7764-7765.

2. Amaro RE, Cheng X, Ivanov I, Xu D, McCammon JA (2009) Characterizing loop dynamics and ligand recognition in human- and avian-type influenza neuraminidases via generalized born molecular dynamics and end-point free energy calculations. J Am Chem Soc 131: 4702-4709.
